# On the structural basis and design guidelines for type II topoisomerase-targeting anticancer drugs

**DOI:** 10.1093/nar/gkt828

**Published:** 2013-09-14

**Authors:** Chyuan-Chuan Wu, Yi-Ching Li, Ying-Ren Wang, Tsai-Kun Li, Nei-Li Chan

**Affiliations:** ^1^Institute of Biochemistry and Molecular Biology, College of Medicine, National Taiwan University, Taipei 100, Taiwan, ^2^Institute of Biochemistry, College of Life Sciences, National Chung Hsing University, Taichung 402, Taiwan, ^3^Department and Graduate Institute of Microbiology, College of Medicine, National Taiwan University, Taipei 100, Taiwan and ^4^Center for Biotechnology, National Taiwan University, Taipei 106, Taiwan

## Abstract

Type II topoisomerases (Top2s) alter DNA topology via the formation of an enzyme–DNA adduct termed cleavage complex, which harbors a transient double-strand break in one DNA to allow the passage of another. Agents targeting human Top2s are clinically active anticancer drugs whose trapping of Top2-mediated DNA breakage effectively induces genome fragmentation and cell death. To understand the structural basis of this drug action, we previously determined the structure of human Top2 β-isoform forming a cleavage complex with the drug etoposide and DNA, and described the insertion of drug into DNA cleavage site and drug-induced decoupling of catalytic groups. By developing a post-crystallization drug replacement procedure that simplifies structural characterization of drug-stabilized cleavage complexes, we have extended the analysis toward other structurally distinct drugs, *m*-AMSA and mitoxantrone. Besides the expected drug intercalation, a switch in ribose puckering in the 3′-nucleotide of the cleavage site was robustly observed in the new structures, representing a new mechanism for trapping the Top2 cleavage complex. Analysis of drug-binding modes and the conformational landscapes of the drug-binding pockets provide rationalization of the drugs’ structural-activity relationships and explain why Top2 mutants exhibit differential effects toward each drug. Drug design guidelines were proposed to facilitate the development of isoform-specific Top2-targeting anticancer agents.

## INTRODUCTION

Type II topoisomerases (Top2s) are a group of essential enzymes that are capable of passing two duplex DNA segments through each other; such an activity allows these enzymes to manipulate DNA topology and resolve geometric DNA entanglements that originate from cellular DNA transactions (1,2). Each cycle of Top2-catalyzed DNA topological transformation constitutes the following steps in sequential order: binding one DNA duplex to the enzyme’s DNA-binding and cleavage core (Top2^core^); producing a double-strand break on the bound DNA via the formation of a covalent enzyme-DNA adduct termed a Top2 cleavage complex (Top2cc); capturing and transporting a second DNA duplex through the transiently formed break; and resealing the cleaved DNA backbones followed by enzyme resetting [for recent reviews and development on the catalytic mechanism of Top2, see (2–5)].

The DNA cleavage activity of Top2 is accomplished by a transesterification reaction between a pair of dyad-related tyrosine residues and two phosphodiester bonds 4-bp apart on opposite DNA strands. The resultant 5′-phosphotyrosyl linkage is energetically equivalent to a phosphodiester bond; therefore, a nucleophilic back-attack by the displaced 3′-OH readily initiates the reverse reaction to restore the nicked DNA backbone. Owing to the reversible nature of transesterification chemistry, the enzyme-linked broken DNA ends embedded within the Top2cc are normally rejoined faithfully and do not present a threat to cell viability. However, when the DNA resealing step is inhibited, the arrested Top2cc may be converted into an irreversible and cytotoxic double-strand DNA break upon its collision with DNA-tracking machineries (6). Many clinically active anticancer and antimicrobial drugs exploit this latent jeopardy of Top2 and exert their cell-killing effects by trapping and accumulating the usually short-lived Top2cc, leading to massive genome fragmentation followed by cell death (6–8).

**Figure 1.**
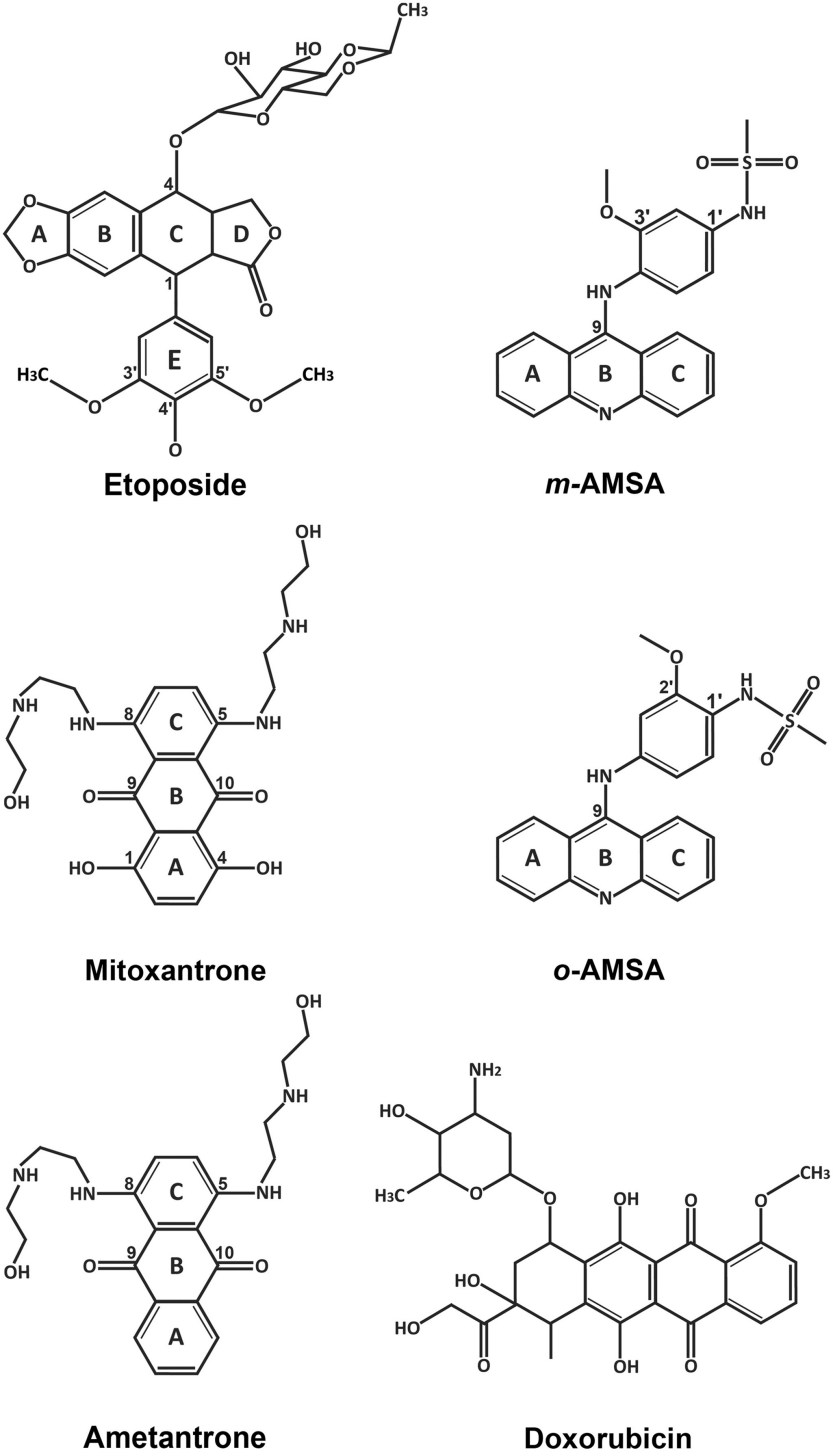
Chemical structures of compounds described in this study.

Drugs targeting eukaryotic Top2s, including etoposide (VP-16), amsacrine (*m*-AMSA), mitoxantrone, doxorubicin and their derivatives (Figure 1), are potent inducers of DNA damage and are widely used in anticancer chemotherapy (8,9). To understand the structural basis of Top2 inhibition by anticancer drugs, we previously solved the structure of the DNA-binding and cleavage core of human Top2 β-isoform (hTop2β^core^) forming a ternary cleavage complex with the drug etoposide and DNA (10). The observed insertion of etoposide at the DNA cleavage site and between the protein-linked 5′-phosphate (of the +1 nucleotide) and displaced 3′-OH (of the −1 nucleotide) reveal that the drug maintains DNA in a cleaved state by physically blocking the religation reaction. Structures of fluoroquinolone antibiotic-bound cleavage complexes of Top IV, a bacterial type II enzyme, further support that cleavage site-specific drug intercalation as a general mechanism of inhibition used by Top2-targeting drugs (11–13). In addition to stacking between the two base pairs flanking the cleavage site, etoposide interacts extensively with protein residues on the DNA major and minor groove sides. The elucidation of protein–drug interactions offers structure-based explanations for the binding specificity of etoposide toward the DNA cleavage site, the drug’s structure–activity relationships and the molecular basis of drug-resistant mutations (10,11). An etoposide-induced detachment of the catalytic Mg^2+^ from the 5′-phosphate suggests another approach by which the DNA religation reaction may be suppressed (10).

Although the production of enzyme-mediated DNA breaks represents a common mechanistic theme for Top2-targeting anticancer drugs, it has remained unknown how other structurally distinct drugs are similarly effective in stabilizing the Top2cc. In addition, therapeutic side effects associated with currently available drugs and the emergence of drug-resistant cancer cells call for new antineoplastic agents (14–16), whose development will benefit from a better appreciation of the structural plasticity of drug-binding pockets. Here, we present structural analyses of the Top2cc stabilized by two clinically active anticancer drugs, *m*-AMSA and mitoxantrone. *m*-AMSA is an acridine and has been used in chemotherapies for leukemia for decades (17–19); mitoxantrone is an anthracenedione that is mainly used in treating breast cancer, leukemia, lymphoma and prostate cancer (16). By revealing distinct and drug-specific sets of interactions that stabilize the bound drugs, this work addresses the structure–activity relationships involved and enables the rationalization and prediction of drug-resistant mutations. Moreover, drug-induced structural changes in DNA further our understanding of how the reversal of Top2-mediated DNA cleavage can be inhibited. Guidelines for designing Top2-targeting agents are proposed based on the available structures, and a post-crystallization drug replacement procedure suitable for the structural determination of drug-stabilized Top2cc has been established to facilitate drug development.

## MATERIALS AND METHODS

### Crystallization and post-crystallization drug replacement

The crystallization condition and the 20-bp DNA duplex used for growing crystals of the hTop2β^core^-DNA-etoposide ternary complex were described previously (10). To obtain Top2cc crystals stabilized by a different anticancer drug (*m*-AMSA, mitoxantrone or ametantrone), etoposide was first soaked out by transferring the etoposide-bound crystals into a substitute mother liquor containing 100 mM magnesium acetate, 50 mM 2-(N-morpholino)ethanesulfonic acid (pH 5.8) and 30% 2-methyl-2,4-pentanediol for 16 h. Next, *m*-AMSA, mitoxantrone or ametantrone were introduced into the drug-free crystals by adding 1 mM of the respective drug (solubilized in dimethyl sulfoxide) to the substitute mother liquor and soaked for another 16 h before looping and flash-freezing in liquid nitrogen for data collection.

### Structure determination

The diffraction data for the hTop2β^core^-DNA-*m*-AMSA, hTop2β^core^-DNA-mitoxantrone, hTop2β^core^-DNA-ametantrone and hTop2β^core^-DNA complexes were collected at NSRRC, Taiwan (beamlines BL13B1 and BL13C1). All diffraction data were processed using the HKL2000 program suite (20). All structures were solved by directly submitting the respective diffraction data sets to rigid body refinement using the drug-free structure of the hTop2β^core^-DNA-etoposide complex (PDBid: 3QX3) as the starting model. The resulting m*F*_o_-D*F*_c_ difference electron density maps of the *m*-AMSA-, mitoxantrone- and ametantrone-soaking in structures showed the presence of the respective drugs at the two DNA cleavage sites, and the structures of the drugs were built into the difference density using Coot (21). All structures then underwent rounds of manual model rebuilding and refinement using Coot and PHENIX (22). All figures were generated using Pymol (23).

## RESULTS AND DISCUSSION

### X-ray crystallography

**Figure 2.**
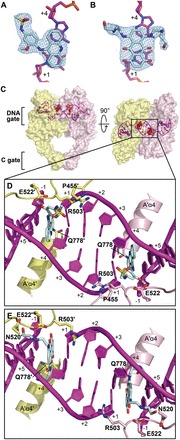
Anticancer drugs *m*-AMSA and mitoxantrone inhibit human Top2 by inserting into enzyme-mediated DNA cleavage sites. (**A** and **B**) The final 2m*F*_o_-D*F*_c_ maps (contoured at 1.5σ; in blue meshes) of bound *m*-AMSA and mitoxantrone, respectively. (**C**) Orthogonal views of the hTop2β^core^-DNA-*m*-AMSA ternary complex. The dimeric hTop2β^core^ protein is shown as a surface representation and is colored according to polypeptide chain. DNA is shown in cartoon and purple. Two *m*-AMSA molecules at each DNA cleavage site are shown as red spheres. (**D** and **E**) The enclosed region of (C) is shown in enlarged views to illustrate the interfacial binding modes of *m*-AMSA and mitoxantrone in the respective drug-stabilized hTop2cc. Drug molecules (in cyan) and side chains of selected drug-contacting residues are shown as sticks. Labels belonging to the second protein monomer are flagged by a prime. Positive and negative numbers designate nucleotides downstream and upstream of the scissile phosphate, respectively, with the +1 nucleotide forming a phosphotyrosyl linkage with the active site tyrosine.

Our group has previously reported the method by which high-quality crystals of etoposide-stabilized Top2cc can be produced for X-ray diffraction analysis (10). To elucidate the molecular basis of Top2 inhibition by other structurally distinct classes of Top2-tageting anticancer drugs (Figure 1), we prepared crystals of *m*-AMSA and mitoxantrone-stabilized Top2cc by replacing the bound etoposide with the respective drugs. Specifically, crystals of the hTop2β^core^-DNA-etoposide ternary complex were first transferred to a drug-free stabilization buffer to release the bound etoposide molecules from these crystals. Other anticancer drugs were then introduced by placing the pre-soaked crystals in a solution that contained the different drug to produce new drug-stabilized Top2cc crystals. Such post-crystallization treatments have allowed the structures of ternary Top2cc stabilized by *m*-AMSA or mitoxantrone to be successfully determined at 2.7 and 2.55 Å resolution, respectively; the binding of new drug molecules, the formation of a pair of 5′-phosphotyrosyl covalent linkages and the presence of a double-strand DNA break are unambiguously defined in the resulting electron density maps (Figure 2A and B; Supplementary Figure S1). Data collection and structural determination statistics are summarized in Table 1.

**Table 1.** Summary of crystallographic analysis

Structure	hTop2β^core^-DNA- *m*-AMSA ternary complex	hTop2β^core^-DNA- mitoxatrone ternary complex	hTop2β^core^-DNA- ametantrone ternary complex	hTop2β^core^-DNA binary complex
Space group	*P2_1_*			
Unit cell dimensions				
*a, b, c* (Å)	80.9, 175.8, 93.1	80.5, 176.6, 93.7	80.7, 176.4, 93.6	79.9, 176.4, 94.2
β (degrees)	113.6	111.5	112.0	112.1
Data collection				
Wavelength (Å)	0.97622	0.97622	1.00000	0.97622
Resolution range (Å)	30.0–2.70 (2.75–2.70)	30.0–2.55 (2.59–2.55)	30.0–2.70 (2.75–2.70)	30.0–2.30 (2.34–2.30)
Observed reflections	188 131	281 721	242 273	311 778
Unique reflections	63 644	78 830	65 943	103 829
Completeness (%)	97.4 (98.8)	99.4 (97.3)	99.1 (91.7)	97.4 (99.0)
Multiplicity	3.0	3.6	3.7	3.1
Mean〈*I/σI*〉	10.4 (2.6)	12.1 (2.5)	12.4 (2.5)	13.4 (2.6)
*R_sym_* (%)^a^	0.08 (0.47)	0.07 (0.49)	0.09 (0.42)	0.06 (0.49)
Refinement				
Resolution range (Å)	28.23–2.70 (2.79–2.67)	27.94–2.55 (2.64–2.55)	27.50–2.70 (2.79–2.70)	27.31–2.30 (2.38–2.30)
Total reflections	65 466	79 377	66 712	106 916
Unique reflections	63 613 (6320)	78 798 (7607)	65 912 (6012)	103 794 (10 147)
Completeness (%)	97.17 (96.30)	99.27 (96.43)	98.80 (90.73)	97.08 (95.21)
No. of reflection in				
working set	63 605	78 789	65 898	99 340
test set	3235	3956	3340	4954
Mean〈*I/σI*〉	10.18 (3.13)	12.52 (2.66)	11.22 (3.03)	11.32 (2.68)
Wilson B-factor	42.71	42.58	43.28	39.08
*R*_crys_ (%)^b^	0.16	0.16	0.17	0.18
*R*_free_ (%)^b^	0.21	0.21	0.22	0.22
Number of atoms	12 054	12 243	12 186	12 461
Macromolecules	11 566	11 614	11 639	11 753
Ligands	62	70	126	7
Waters	426	559	421	701
Protein residues	1363	1369	1368	1375
r.m.s. deviation from ideal				
Bond lengths (Å)	0.008	0.008	0.008	0.007
Bond angles (degrees)	1.33	1.14	1.16	1.07
Ramachandran analysis^c^				
Outliers (%)	0.3	0.23	0.2	0.15
Favored (%)	96	96	96	97
Clashscore	8.25	8.00	9.33	14.06
Average B-factor	31.20	33.30	44.80	44.20
Macromolecules	31.30	33.40	45.20	44.20
Solvent	28.00	31.20	38.90	44.70

Statistics for the highest-resolution shell are shown in parentheses.

^a^*R*_sym_ = (Σ|*Ihkl* -〈*I*〉)/(Σ*Ihkl*), where the average intensity 〈*I*〉 is taken overall symmetry equivalent measurements, and *Ihkl* is the measured intensity for any given reflection.

^b^*R*_cryst_ = (Σ||*F_o_*| - *k*|*F_c_*||)/(Σ|*F_o_*|). *R*_free_ = *R*_cryst_ for a randomly selected subset (5%) of the data that were not used for minimization of the crystallographic residual.

^c^Categories were defined by PHENIX (22).

### Structural basis of Top2 inhibition by the anticancer drugs *m*-AMSA and mitoxantrone

**Figure 3.**
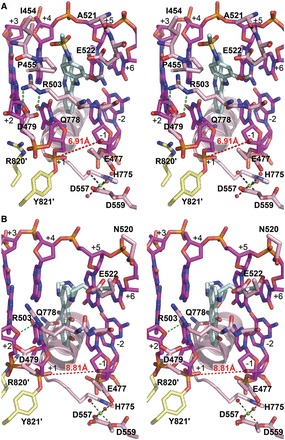
Detailed views of drug-binding sites. Stereo representations of the binding sites for *m*-AMSA (**A**) and mitoxantrone (**B**). Drugs and DNA are shown as cyan and purple sticks, respectively. Two hTop2β^core^ monomers are colored differently. Labels belonging to the second monomer are flagged by a prime. Forces that stabilize the R503 side chain in each structure are indicated by green dashed lines. Mg^2+^ and water molecules are shown as green and red spheres, respectively. The distance between the Y821′-linked scissile phosphate and the 3′-OH are indicated.

The binding of *m*-AMSA and mitoxantrone between the +1/+4 and −1/+5 DNA base pairs immediately flanking the cleavage sites reveals that, despite significant differences in chemical structure (Figure 1), these two drugs share a similar mechanism of Top2 inhibition with etoposide (Figure 2C–E) (10). The cleavage site-specific drug insertion physically interferes with the stacking between the +1/+4 and −1/+5 base pairs and maintains the separation of the 3′-OH (of the −1 nucleotide) and the enzyme-linked 5′-phosphate (of the +1 nucleotide) by 7 ∼ 8 Å (Figure 3), which effectively traps the Top2cc by preventing the religation reaction. Moreover, compared with the quaternary conformation adopted by two drug-free Top2-DNA binary complexes with a catalytically active DNA cleavage center (24,25), it has been suggested that the etoposide-induced separation of the cleaved DNA ends is accompanied by the two dyad-related Top2 monomers sliding away from each other along their respective H3 helices (10). Similar types of drug-promoted quaternary conformational changes were also observed in the *m*-AMSA and mitoxantrone-bound structures, which are expected to further discourage the religation reaction by disrupting the *in trans*-assembled Top2 cleavage center (Supplementary Figure S2).

**Figure 4.**
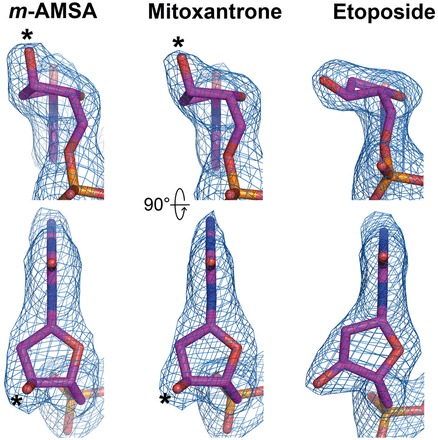
The unbiased difference electron density maps (m*F*_o_-D*F*_c_, contoured at 4.5σ) of the −1 nucleotides are shown to validate the ring puckering change in the deoxyribose in the presence of the respective drugs. The *m*-AMSA- and mitoxantrone-stabilized axial positions of 3′-OH are indicated by black stars.

A notable difference between the parental etoposide-stabilized and the two new Top2cc structures is a switch of deoxyribose ring puckering of the −1 and +5 nucleotides from the 3′-endo to the 2′-endo configuration (Figure 4). The potential significance and validity of this finding are further corroborated by the observation of an identical structural transition in the two crystallographically independent DNA breaks in the asymmetric unit. In addition, three extra crystallographic approaches were used to provide additional support for the change in ring puckering. First, we deliberately modeled the −1 and +5 nucleotides of the *m*-AMSA- and mitoxantrone-bound structures to adopt the 3′-endo configuration, as observed in the etoposide-bound structure (10); rounds of structural refinement were then applied using the refinement module of PHENIX (22). In both cases, the converged models reverted to the 2′-endo configuration. A reciprocal test also confirmed the robustness of the tendency for these nucleotides to take the 3′-endo configuration in the presence of etoposide. Secondly, we removed the −1 and +5 nucleotides from the three Top2cc structures, and the partial models were subjected to structural refinement using PHENIX. Unbiased m*F*_o_-D*F*_c_ electron density maps calculated using the converged partial models clearly show features that agree with the switching in ring puckering for the omitted nucleotides on drug replacement (Figure 4). Finally, structural analyses performed on the drug-free and etoposide-reintroduced crystals revealed that the −1 and +5 nucleotides adopt the 3′-endo configuration in both structures (Supplementary Figure S3), which effectively rules out the post-crystallization handling procedures as the cause for the change in ribose puckering. Taken together, these findings indicated that the formation of 2′-endo riboses is induced specifically by *m*-AMSA and mitoxantrone; the alternation of R503 rotamer conformation resulted from drug-replacement and the more pronounced stacking between these two drugs and the flanking base pairs are possible causes for the switching in deoxyribose ring puckering (Supplementary Figure S4).

Accompanying the switch in ribose puckering from the 3′-endo to the 2′-endo conformation, the equatorially placed 3′-OH of the −1 nucleotide rearranges to take an axial position, which constrains the lone pair electrons on the oxygen of 3′-OH to point away from the 5′-phosphate. Such a structural transition likely perturbs the alignment between the nucleophile (the 3′-OH) and the electrophile (the 5′-phosphotyrosine) and may thus suppress the religation reaction. Collectively, the currently available structures of drug-stabilized Top2cc suggest the insertion between cleaved DNA ends, the decoupling of catalytic groups and the axial repositioning of the 3′-OH of the −1 nucleotide as three possible mechanisms by which the religation reaction can be inhibited.

### Cleavage site-specific targeting by *m*-AMSA and mitoxantrone

Whereas the insertion of their polycyclic aglycone cores between the +1/+4 and −1/+5 base pairs appears to be a major and common mechanistic theme used by drugs targeting the Top2cc, it is well known that the DNA-intercalating potential alone is not sufficient for conferring Top2-poisoning activity. For example, the DNA intercalator ethidium bromide inhibits Top2 activity at higher concentrations but fails to induce Top2cc formation (26), indicating that the intercalation may not be cleavage site-specific and that the inhibitory effect likely results from a reduced association between Top2 and DNA owing to interference from randomly intercalated ethidium bromide. To effectively trap the sparsely populated and transiently formed Top2cc during the catalytic cycle, stabilizing interactions between the protein and the bound drug are required to arrest the drug in the Top2-mediated DNA cleavage site. By revealing the extensive networks of protein–drug interactions, our structures suggest how the cleavage site-specific binding by *m*-AMSA and mitoxantrone is achieved. As shown in Figures 2D and 3A, the planar acridine chromophore of *m*-AMSA (rings A∼C in Figure 1) inserts into the cleavage site to maintain the separation of the two flanking base pairs, and the bulky methanesulfon-*m*-anisidide head group protrudes toward the DNA minor groove to mediate specific drug–protein contacts. The anilino moiety packs against the aliphatic part of the R503 side chain, and the methanesulfonamide moiety is anchored by forming a hydrogen bond with E522 and van der Waals interactions with I454, P455, P504, A521 and E522.

**Figure 5.**
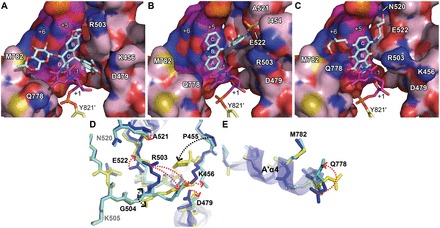
Conformational landscapes of the drug-binding pockets for mammalian Top2-targeting anticancer drugs. (**A–C**) Surface/stick representations of the etoposide-, *m*-AMSA- and mitoxantrone-binding sites, respectively. Selected DNA base pairs (purple) and protein (pink) residues are shown as surface representations and are labeled using white letters. Two hTop2β^core^ monomers are colored differently. Drugs (cyan) and the Y821′-conjugated +1 thymidines are shown as sticks. Labels belonging to the second monomer are flagged by a prime. Cyan letters designate polycyclic ring labeling of drugs. (**D** and **E**) Superposition of residues 445–731 and residues 762–821 in hTop2β of the drug-stabilized hTop2cc structures to show structural differences in the minor groove and major groove drug-binding pockets, respectively. Selected residues of hTop2cc structures stabilized by etoposide (blue), *m*-AMSA (yellow) and mitoxantrone (cyan) are shown as sticks. Side chain and main chain variations are indicated by red and black arrows, respectively. Residues that exhibit no structural change under drug binding are labeled in gray.

Similarly, mitoxantrone uses its polycyclic aromatic dihydroxy-anthraquinone moiety (rings A∼C in Figure 1) to intercalate into the DNA cleavage site and physically blocks the religation reaction (Figures 2E and 3B). In contrast to *m*-AMSA, whose acridine moiety functions exclusively in DNA-intercalation and mediates no direct interactions with the protein, the polycyclic core of mitoxantrone plays dual roles, as the hydroxyl and carbonyl groups of rings A and B are also involved in hydrogen bonds with G504 on the minor groove and Q778 on the major groove side. The two structurally identical hydroxylalkylamino arms that attach symmetrically to the C-ring (via the C5 and C8 carbon atoms) project toward the DNA major and minor grooves, respectively, embracing the +5 guanine base from both sides. Additional interactions that stabilize the bound mitoxantrone are mainly provided by the minor groove-placed hydroxylalkylamino arm, which fits into a crevice located between the protein and the DNA and forms multiple hydrogen bonds with the side chains of N520 and E522 and the main-chain carbonyl group of R503. In contrast, the arm in the major groove is more solvent-exposed and only a single hydrogen bond is present between the drug and the protein. We noted that an earlier nuclear magnetic resonance-based study of the (protein-free) mitoxantrone-RNA binary complex placed both alkylamino arms in the major groove (27); however, our modeling analysis indicates that a similar type of binding is not feasible in the context of the Top2 cleavage complex owing to steric conflicts between the drug and protein residues in both DNA minor and major groove. Comparing the structures of Top2cc stabilized by different drugs clearly revealed that the binding of each drug in the cleavage site is mediated by a distinct and drug-specific set of interactions (Figure 5A–C).

### Structure–activity relationships and the structural basis of drug resistance

Because the *m*-AMSA- and mitoxantrone-stabilized Top2cc structures were obtained by post-crystallization ligand replacement rather than direct crystallization, the validity and pharmacological relevance of these new structures must be carefully assessed. Toward this end, we examined whether the structure–activity relationships and drug-resistant mutations known for each drug can be fully accounted for by the observed interaction patterns and drug-binding modes. Among the residues involved in *m*-AMSA binding (Figure 3A), E522 appears to play a key role with its side-chain carboxylate forming a salt bridge to the amine group of the 1′-methanesulfon substitution, and its Cβ-Cγ-Cδ moiety contacting the 3′-methoxy group and the C2′ atom of the aniline ring (Figure 3A). This set of stabilizing interactions readily explains the impairment of drug activity on the removal of the 1′- and 3′-substitutions from the methanesulfon-*m*-anisidide head group (19), which is expected to substantially weaken drug binding. The *m*-AMSA-bound structure also addresses why *o*-AMSA (Figure 1) is essentially inactive at inducing Top2-mediated DNA breakage compared with the high potency exhibited by *m*-AMSA (26); steric clashes between the methoxy group and the E522 side chain would be produced when shifting the methoxy group from the meta (3′, as in *m*-AMSA) to the ortho position (2′, as in *o*-AMSA). Therefore, despite the fact that the two AMSA stereoisomers display comparable DNA-intercalating activity (26), *o*-AMSA and other AMSA analogs with ortho-substitutions are incompatible with the minor groove drug-binding pocket and are thus inefficient at stabilizing Top2cc (19). Intriguingly, when detached from the acridine chromophore, the methanesulfon-*m*-anisidide head group of *m*-AMSA alone is capable of trapping Top2cc at high concentrations (19). We suspect that by occupying the minor groove binding pocket, the anilino head group may cause the −1 nucleotide to adopt the less-reactive 2′-endo conformation, as noted in the *m*-AMSA-bound structure (Figure 4), thereby giving rise to DNA intercalation-independent Top2-poisoning activity.

The identification of a glutamate-to-lysine mutation at E522 (E522K) as being resistant to *m*-AMSA and other acridine derivatives (28) also agrees with our structural observation; most likely, the longer lysine side chain would clash with the anilino ring to disfavor drug binding. Given that E522 does not interact directly with etoposide (Figure 5A) (10), the present study establishes the importance of this residue for the formation of drug-stabilized Top2cc. Interestingly, the E522K mutation displayed hypersensitivity toward etoposide (28), suggesting that the mutation-induced changes in shape and polarity of the minor-groove drug-binding pocket disturb *m*-AMSA binding but facilitate the binding of etoposide. The observed stabilizing interactions also explain why mutations at R503, another major drug-contacting residue, or the nearby P501 and L502 residues may weaken the protein–drug interactions and thus result in drug resistance (10,29).

Mitoxantrone and ametantrone are structurally closely related derivatives of anthracycline (Figure 1), both being effective anticancer agents with reduced cardiotoxicity compared with their parent compounds (30). While exhibiting similar DNA-intercalating activities (31), however, the Top2-poisoning and antitumor activities of ametantrone are significantly lower than mitoxantrone (32,33), likely because the A ring of ametantrone is not hydroxylated. Using the post-crystallization soaking procedure described above, the structure of ametantrone-bound Top2cc was determined (Table 1 and Supplementary Figure S5). Except for a difference between ring A-mediated interactions and minor adjustments of the nearby protein moieties, these two anthracenedione-stabilized structures are essentially indistinguishable, indicating that a loss of 1- and 4-hydroxyl groups compromises drug function mainly by reducing the number of drug–protein contacts. Additionally, interactions between the hydroxylalkylamino arms and the protein address why modifications of these two branching moieties affect drug efficacy (Figure 5C and Supplementary Figure S5C) (34). Taken together, these findings indicate that the new structures are pharmacologically relevant and that the reported ligand-replacement procedure for the structural characterization of drug-stabilized Top2cc may benefit the structure-based development of new Top2-targeting drugs.

### Conformational landscapes of the drug-binding pocket

The three drug-stabilized Top2cc structures reveal a substantial repositioning of drug-contacting residues located on the DNA minor groove side on the binding of various drugs (Figure 5); this finding explains how structurally distinct drugs are accommodated within the protein–DNA interface. The observed structural rearrangements are centered on R503 and are tightly correlated with the location of the minor groove-protruding moiety of each drug. For etoposide, the E-ring faces the cleaved +1 nucleotide and interacts with the nearby K456 and D479 residues, causing the R503 side chain to swing toward the +4 and +5 nucleotide of the opposing strand (Figure 5A). In contrast, both the anilino head group of *m*-AMSA and the alkylamino arm of mitoxantrone extend into the pocket formed between the +5 nucleotide and flanking residues including E522 (Figure 5B and C), which drives R503 to the vicinity of the +1 nucleotide. Therefore, two distinctive but mutually exclusive regions on the minor groove side of the protein–DNA interface are recognized as crucial for drug binding. Although residues located in the minor groove exhibit notable structural variations on drug replacement (Figure 5D), few changes were detected for the primary drug-interacting residues on the major groove side (Figure 5E), consistent with the notion that the major groove pocket is sufficiently spacious to accommodate drug moieties of different sizes and shapes (10). By demonstrating the magnitude of the structural changes associated with drug-contacting residues, together, these structures reveal a set of conformational landscapes of the drug-binding sites, which may facilitate *in silico* design of new Top2-targeting agents.

Superimposition of the three drug-stabilized Top2cc structures also shows that structural differences caused by drug exchange are mainly owing to side-chain rearrangements and are confined to the immediate vicinity of the drug-binding pockets, and the approach of P455 toward the anilino head group of *m*-AMSA is the only evident main-chain displacement (Figure 5D and E). Because P455 is located within a flexible loop that is detached from the core protein domains, this movement should not significantly affect the overall structure. The highly localized nature of these structural changes explains why drug replacement can be achieved without disrupting crystal packing. Despite the usefulness of the soaking procedure in illustrating the binding modes of *m*-AMSA and mitoxantrone, this technique may not be applicable to intercalating compounds with bulky groups facing the DNA minor groove, such as doxorubicin (Figure 1). The structure of a doxorubicin-DNA binary complex suggests a preference for locating the amino sugar and hydroxymethyl ketone groups in the minor groove on the drug’s intercalation into DNA (35). Assuming that this binding mode of doxorubicin dictates and is not affected by the involvement of Top2, then the presence of doxorubicin in the cleavage site would be strongly discouraged owing to severe steric repulsion from the minor groove-flanking residues when the quaternary structure of Top2cc is constrained by the crystal lattice. Indeed, when soaked into the crystal, doxorubicin was found to bind between the +1/+4 and +2/+3 base pairs rather than the cleavage site (Supplementary Figure S6 and Table S1). Given this potential limitation, we suggest that the reported soaking procedure for drug exchange would be most suitable for those Top2-targeting agents whose binding (at the cleavage site) can be accommodated by local side-chain rearrangements.

### Guidelines for designing new Top2-targeting agents

**Figure 6.**
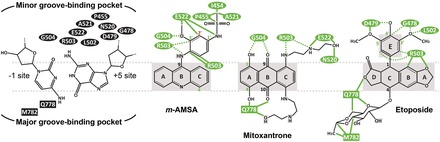
The binding modes of Top2-targeting agents. Chemical structures of −1/+5 base pairs and drugs are shown to illustrate the orientation of drug binding. Drug-contacting residues in the minor (in oval-shade) and major (in square-shade) groove-binding pockets are indicated. For each drug, atoms involved in drug–DNA interactions are shaded in gray. The interactions mediated by side chain and main chain atoms are shown as green solid and dashed lines, respectively. Atoms involved in substitutions that increase the drug’s potency are labeled in green. Substitutions that abolish drug action are labeled in red.

Despite the proven efficacy of Top2-targeting drugs in anticancer chemotherapy, their long-term use is known to impose potential risks in patients owing to undesirable side effects (16,36). Recent studies suggest that the disadvantages associated with these drugs may be partially alleviated by more specific targeting of hTop2α without the simultaneous poisoning of hTop2β, which may induce chromosome rearrangements and lead to therapy-related leukemia (14,37). Therefore, the development of hTop2α-specific targeting drugs may benefit cancer treatment. Based on the mechanisms of action, structure–activity relationships and chemical features common to the three distinct classes of anticancer drugs and by recognizing that variations in the drug-contacting residues between hTop2α and hTop2β are located in the major groove drug-binding pocket (Figure 6), we have formulated three general guidelines for developing isoform-specific targeting agents as follows: (i) A polycyclic aromatic core is required to facilitate drug intercalation into the DNA cleavage site. (ii) Attaching a branching moiety (or moieties) that fits between the protein and DNA minor groove to the aromatic core enhances the drug’s affinity and specificity toward the cleavage site. (iii) Introducing a branching moiety to the aromatic core that extends toward the DNA major groove aids in mediating isoform-specific interactions.

The application of guidelines (i) and (ii) should be sufficient for constructing a Top2-targeting agent, as illustrated by *m*-AMSA, which lacks a major groove-protruding group (Figures 5B and 6). However, because residues forming the minor groove drug-binding pocket are entirely conserved between the two human isoforms, exploiting guideline (iii) is required to enable a compound to target hTop2α more specifically. Key differences on the major groove side include the replacement of Q778 and A816 (in hTop2β) by M762 and S800, respectively, in the α-isoform (10,25). We speculated that M762 and the adjacent M780 present a hTop2α-specific hydrophobic surface and would interact more strongly with a nonpolar major groove-protruding moiety (10). It has also been suggested that these two juxtaposed methionine residues in hTop2α may exhibit higher reactivity toward platinum compounds compared with hTop2β, and the relevance of a more distantly located variation (S800/A816) in distinguishing isoforms may also be evaluated (25). Moreover, as an ideal drug should target hTop2α efficiently while sparing hTop2β to the utmost, therefore, we envision a new Top2-targeting drug with reduced drug–protein interactions in the minor groove binding pocket but instead relying more heavily on its major groove-protruding group(s) for mediating cleavage site-specific binding. Given the aforementioned strategies for exploiting the differences between hTop2α and hTop2β, the development of hTop2α-specific drugs should be an achievable goal.

## CONCLUSION

In this work, we have determined the high-resolution crystal structures of hTop2β cleavage complexes stabilized by the anticancer drugs *m*-AMSA and mitoxantrone. These structures not only advance our understanding of the inhibitory mechanisms of Top2-targeting anticancer drugs and illustrate the conformational landscapes of drug-binding pockets but also lead to the formulation of design guidelines for developing isoform-specific targeting agents. The post-crystallization drug replacement procedure reported here should significantly improve the efficiency of drug development by accelerating the structural analysis of drug-stabilized Top2cc.

## ACCESSION NUMBERS

Atomic coordinates and structure factors have been deposited in the PDB with accession codes 4G0U (hTop2β^core^-DNA-*m*-AMSA ternary complex), 4G0V (hTop2β^core^-DNA-mitoxantrone ternary complex), 4G0W (hTop2β^core^-DNA-ametantrone ternary complex) and 4J3N (hTop2β^core^-DNA binary complex).

## SUPPLEMENTARY DATA

Supplementary Data are available at NAR Online, including [38–40].

## FUNDING

National Science Council [NSC99-2113-M-002-008-MY3 and NSC101-2911-I-002-303-] and the National Research Program for Biopharmaceuticals [NSC101-2325-B-002-049]. Funding for open access: National Taiwan University and National Science Council.

*Conflict of interest statement*. None declared.

## Supplementary Material

Supplementary Data
