# Supplementary material for: On the structural basis and design guidelines for type II topoisomerase-targeting anticancer drugs
Source: Nucleic Acids Res. 2013 Sep 14;41(22):10630–40. doi: 10.1093/nar/gkt828 (PMC3905874; doi:10.1093/nar/gkt828)
Supplement: Supplementary Data [file supp_41_22_10630__index.html]

On the structural basis and design guidelines for type II topoisomerase-targeting anticancer drugs — On the structural basis and design guidelines for type II topoisomerase-targeting anticancer drugs — Supplementary Data 

# On the structural basis and design guidelines for type II topoisomerase-targeting anticancer drugs

## Supplementary Data

files

**Files in this Data Supplement:**

- Supplementary Data - pdf file
