# Supplementary material for: On the structural basis and design guidelines for type II topoisomerase-targeting anticancer drugs
Source: Nucleic Acids Res. 2013 Sep 14;41(22):10630–40. doi: 10.1093/nar/gkt828 (PMC3905874; doi:10.1093/nar/gkt828)
Supplement: Supplementary Data [file supp_gkt828_nar-01496-m-2013-File008.pdf]

Supporting Online Material for

On the Structural Basis and Design Guidelines for Type II Topoisomerase-Targeting Anticancer Drugs

Chyuan-Chuan Wu[1], Yi-Ching Li[1,2], Ying-Ren Wang[1], Tsai-Kun Li[3,4,*] and Nei-Li Chan[1,2,*]

[1]Institute of Biochemistry and Molecular Biology, College of Medicine, National Taiwan University, Taipei 100, Taiwan.

[2]Institute of Biochemistry, College of Life Sciences, National Chung Hsing University, Taichung 402, Taiwan.

[3]Department and Graduate Institute of Microbiology, College of Medicine, National Taiwan University, Taipei 100, Taiwan.

[4]Center for Biotechnology, National Taiwan University, Taipei 106, Taiwan.

***To whom correspondence should be addressed:**

Nei-Li Chan

Institute of Biochemistry and Molecular Biology, College of Medicine, National Taiwan University, Taipei City 100, Taiwan. Tel: 886-2-23562214; Fax: 886-2-23915295

Email: nlchan@ntu.edu.tw

Correspondence may also be addressed to:

Tsai-Kun Li

Department and Graduate Institute of Microbiology, College of Medicine, National Taiwan University, Taipei 100, Taiwan. Tel: 886-2-22123456x88287/88294; Fax: 886-2-23915293

Email: tsaikunli@ntu.edu.tw

This PDF file includes

Supplementary Methods

Supplementary Text

Supplementary Figures S1-6

Supplementary Table S1

Supplementary References

SUPPLEMENTARY METHODS

Preparation of the hTop2β^{core}-DNA-doxorubicin ternary complex crystal by post-crystallization drug replacement

To obtain the hTop2β^{core} cleavage complex stabilized by doxorubicin, etoposide was soaked out by transferring the hTop2β^{core}-DNA-etoposide crystals (1) into a substitute mother liquor containing 30% MPD for 16 hours. Doxorubicin was then introduced by adding 1 mM drug (in DMSO) to the drop containing drug-free crystals. After 16 hours of soaking, crystals were looped and flash-frozen in liquid nitrogen for data collection.

Structure determination

In each deposited structure, missing residues are as follows: hTop2β^{core}-DNA-*m*-AMSA (PDBid: 4G0U): 445-454, 592-647, 694-706 and 1112-1134 in chain A and 445-452, 591-635, 694-705 and 1111-1134 in chain B; hTop2β^{core}-DNA-mitoxantrone (PDBid: 4G0V): 445-451, 592-645, 697-706 and 1112-1134 in chain A and 445-448, 591-636, 694-706, 963-966 and 1111-1134 in chain B; hTop2β^{core}-DNA-ametantrone (PDBid: 4G0W): 445-451, 592-644, 697-706 and 1112-1134 in chain A and 445-448, 593-643, 696-705, 963-966 and 1111-1134 in chain B; hTop2β^{core}-DNA (PDBid: 4J3N): 445-454, 592-639, 697-706 and 1112-1134 in chain A and 445-448, 593-636, 696-705, 963-966 and 1111-1134 in chain B. These regions were omitted from the model.

For the parameters listed in table 1, all non-glycine residues were included for the Ramachandran analysis. Residues which fells in the disallowed region of the Ramachandran plot include: S794 and G868 of chain A and V852 of both chain in hTop2β^{core}-DNA-*m*-AMSA structure; D1101 of chain A and V852 of both chains in hTop2β^{core}-DNA-mitoxantrone structure; D540 of chain A and D1101 of chain B in hTop2β^{core}-DNA-ametantrone structure; D1101 of both chains in hTop2β^{core}-DNA structure. Although are outliers of Ramachandran plot, these residues fit very well to the corresponding electron density.

SUPPLEMENTARY TEXT

Doxorubicin may stabilize the Top2β cleavage complex in a new quaternary conformation.

Doxorubicin and other structurally related anthracycline derivatives represent a group of drugs commonly used to treat various types of cancers, including leukemias, lymphomas, and breast, uterine, ovarian, and lung cancer (2). To understand how doxorubicin interacts and stabilizes Top2cc, we introduced doxorubicin into the drug-free hTop2β^{core}-DNA binary complex crystals and the structure was determined at 2.7 Å resolution (Table S1). Surprisingly, this structure revealed a new drug binding mode distinct from those observed in the etoposide-, *m*-AMSA- and mitoxantrone-bound structures. Rather than binding to both cleavage sites, a single doxorubicin molecule binds asymmetrically within the four-base stagger between the two cleavage sites (Figure S6), which disrupts the usually highly preserved two-fold symmetry associated with Top2 structures. The aglycone moiety of doxorubicin intercalates between the +1/+4 and +2/+3 base pairs and adopts a skewed orientation relative to the two phosphoribosyl backbones while the amino sugar and hydroxymethyl ketone moieties rest in the minor groove. Because there are very few protein-mediated contacts (Figure S6C), the drug's conformation is stabilized mainly by its interactions with DNA. Notably, instead of being sandwiched between two Watson-Crick base pairs, doxorubicin stacks against a Watson-Crick base pair (+2/+3) on one side and a reverse Hoogsteen base pair (+1/+4) on the other. A large repositioning of the +4 adenine base accompanied by a change in ribose ring-puckering likely favors the formation of this non-canonical base pair.

The preference for having the amino sugar and hydroxymethyl ketone groups located in the minor groove, as seen in the structures of doxorubicin-DNA binary complexes (3), may explain why doxorubicin does not bind at the cleavage site where the minor groove is approached by Top2. In the absence of additional quaternary structure changes, the minor groove binding pocket is not spacious enough to accommodate the two appended drug moieties. The protrusion of the amino sugar moiety toward the distal cleavage site prevents the binding of a second doxorubicin molecule within the four-base stagger because the simultaneous presence of two drug molecules at both symmetry-related base pair steps would cause steric clashes between the two amino sugar groups.

Previous studies have revealed a strong Top2 DNA cleavage preference for the −1 nucleotide to be an adenine in the present of doxorubicin, which indicates that doxorubicin would also target the cleavage site for function (4). In

addition, as the bound doxorubicin is mainly stabilized by its interactions with DNA with very few protein-mediated contacts, the new binding mode fails to explain the reported SARs of anthracyclines (2). Thus, we need to be extra cautious about the pharmacological relevance of this structure, and we suspected that the potential "off-site" binding of doxorubicin may be due to steric repulsion between the drug's bulky minor groove-protruding moieties and the flanking residues, considering that the quaternary conformation of Top2 is constrained by the crystal packing. Therefore, despite the usefulness of the soaking method in illustrating the binding modes of *m*-AMSA and mitoxantrone, this technique may not be applicable to compounds such as doxorubicin, due to their bulky groups facing DNA minor groove that can't be accommodated by the protein-embraced DNA cleavage site. And we suspect that doxorubicin would trap the cleavage complex in a more opened conformation due to a wider separation of catalytic groups in the presence of this relatively bulkier drug. With this potential limitation in mind, we suggest that the reported soaking procedure for drug exchange would be most suitable for those Top2-targeting agents whose binding (at the cleavage site) can be accommodated by local side-chain rearrangements.

SUPPLEMENTARY FIGURES

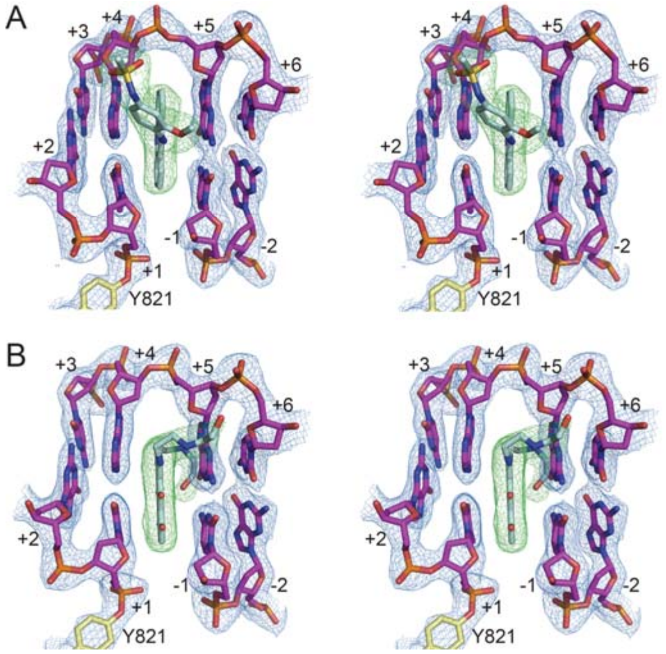

Figure S1. The electron density maps of the bound drugs in the hTop2β^{core}-DNA-*m*-AMSA and hTop2β^{core}-DNA-mitoxantrone ternary complexes. (**A**-**B**) Stereo representations of unbiased mF_o-DF_c difference electron density maps of the drugs show the presence of *m*-AMSA and mitoxantrone in the respective structures. The mF_o-DF_c difference electron density maps (contoured at 3.0σ) of drugs obtained by refinement using the drug-free hTop2β^{core}-DNA structure are shown as green meshes. The final 2mF_o-DF_c maps (contoured at 1.5σ) of selected DNA base pairs and +1 thymidine-conjugated Y821 are shown as blue meshes.

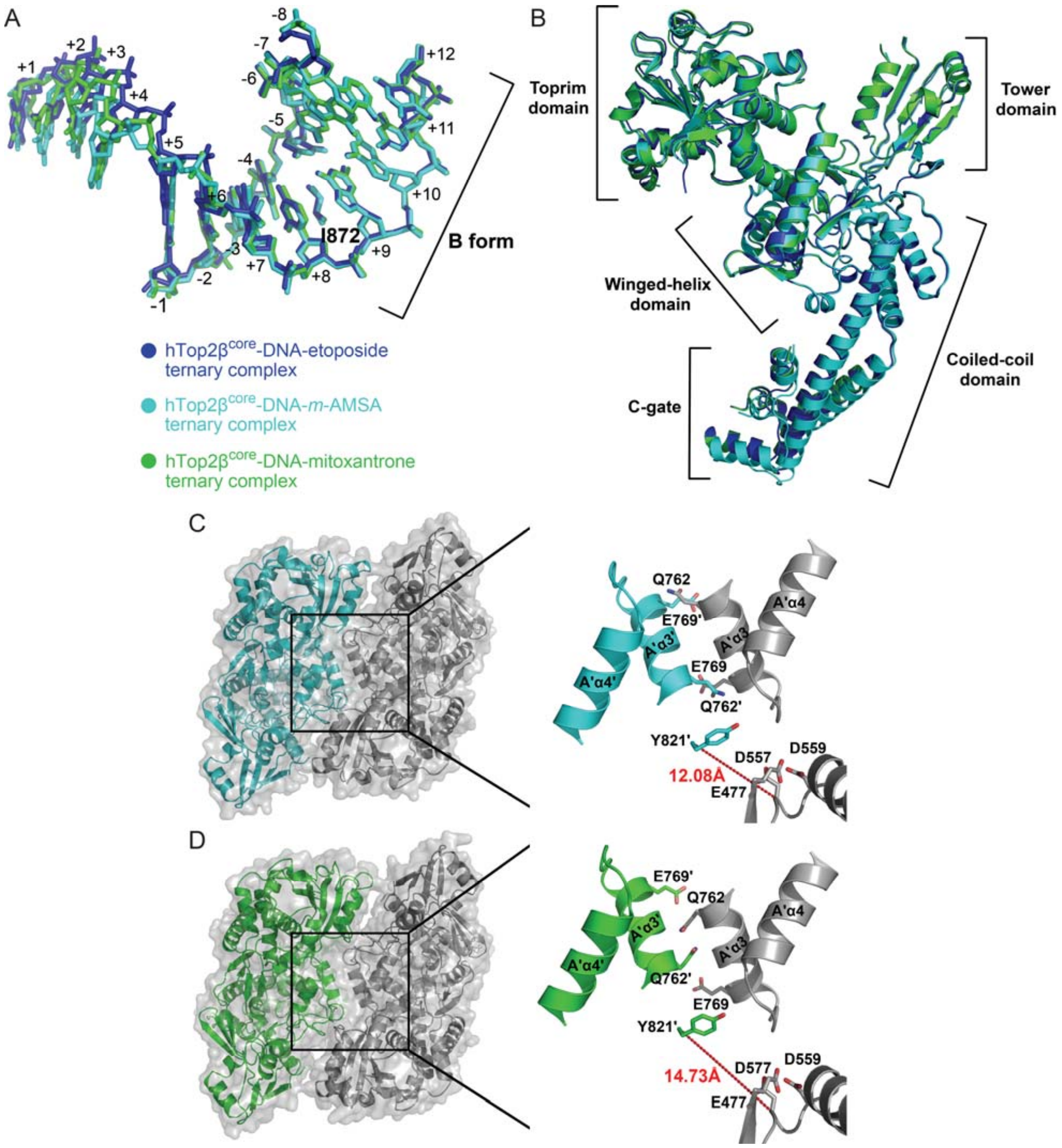

Figure S2. Overall structural comparisons of the drug-stabilized hTop2β cleavage complexes. (**A**-**B**) Superposition of residues 762-821 (including A' helix3, helix4 and the active site Y821) in the three drug-stabilized hTop2β^{core} structure compares the tertiary structure of DNA and protein, respectively. The hTop2β^{core}-DNA-etoposide, hTop2β^{core}-DNA-*m*-AMSA and hTop2β^{core}-DNA-mitoxantrone ternary complexes are colored in blue, cyan and green, respectively. (**C**-**D**) The sliding of the two A' helix3 about the structural dyad indicates a quaternary structural

difference between the hTop2β^{core}-DNA-*m*-AMSA (**B**; in cyan) and hTop2β^{core}-DNA-mitoxantrone (**C**; in green) ternary complexes. Left-hand panels show surface representations of the top views of the two structures. Protein dimers are colored according to their polypeptide chain. For each structure, selected residues from the enclosed region are shown in an enlarged view (right-hand panels) to illustrate the spatial relationship between the catalytic Y821 and the Mg^{2+}-binding acidic triad (E479, D557 and D559); the distance between Y821 and D557 is indicated. Residues belonging to the second monomer are flagged by a prime.

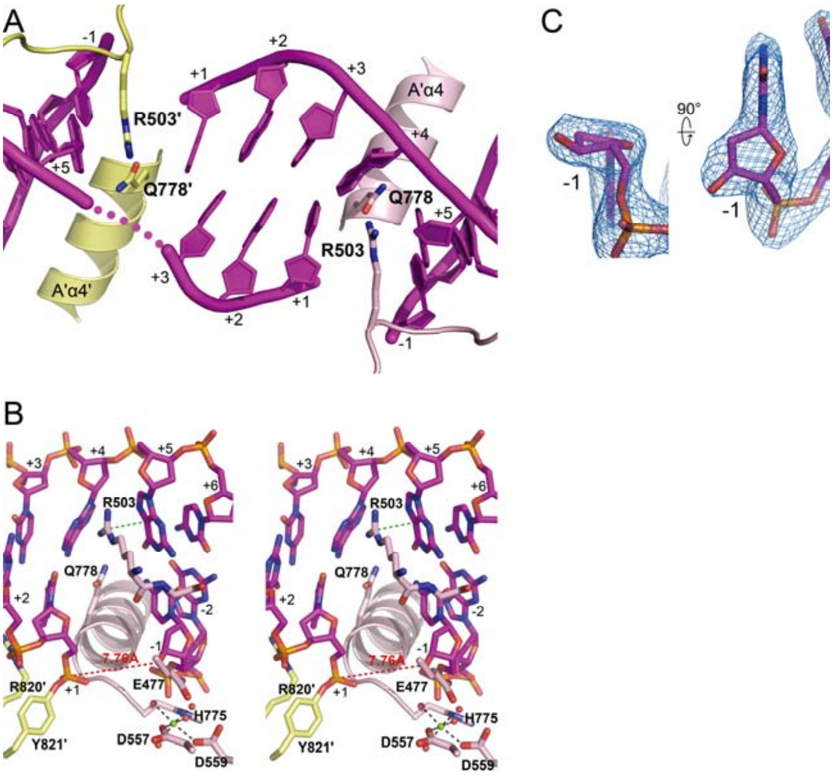

Figure S3. Structural details of the drug-free hTop2β^{core}-DNA cleavage complex. (**A**) The DNA cleavage sites of the hTop2β^{core}-DNA binary complex. Positive and negative numbers designate nucleotides downstream and upstream of the scissile phosphate, respectively, with the +1nucleotide forming a phosphotyrosyl linkage with the active site tyrosine. One of +4 nucleotides is missing and is denoted by a purple dashed line. DNA is shown as purple sticks. The two hTop2β^{core} monomers are colored differently. Labels referring to the second monomer are flagged by a prime. (**B**) Stereo representations of the cleavage site. Force that stabilizes the R503 side chain is indicated by green dashed line. Mg^{2+} and water molecules are shown as green and red spheres, respectively. The distance between the Y821'-linked scissile phosphate and the 3'-OH are indicated. (**C**) The mF_o-DF_c unbiased difference electron density maps (contoured at 4.5σ) of −1 and −2 nucleotides. The drug-free hTop2β^{core}-DNA cleavage complex was obtained by soaking out etoposide from the hTop2β^{core}-DNA-etoposide crystal.

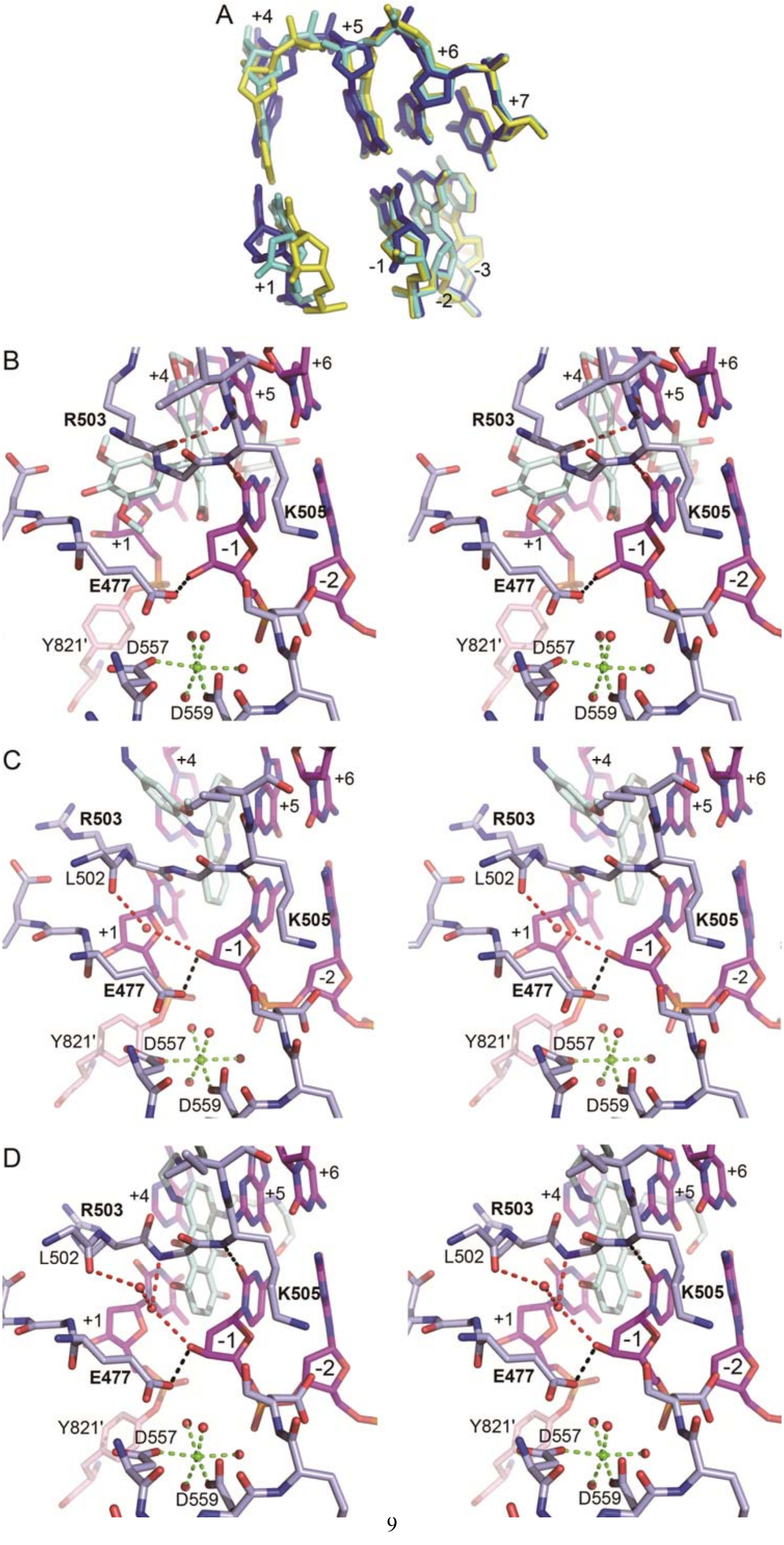
A
+4
+5
+6
+7
+1
-1
-2
-3
B
R503
K505
E477
Y821'
D557
D559
+1
-1
-2
+4
+5
+6
C
R503
L502
K505
E477
Y821'
D557
D559
D
R503
L502
K505
E477
Y821'
D557
D559

Figure S4. Interaction networks around the −1 nucleotide in drug-stabilized hTop2β^{core} cleavage complexes. (**A**) Superimposition of DNA structures from the three drug-stabilized hTop2β^{core} cleavage complexes (from −2/+5 to −8/+12 base pairs with respect to one protomer) to illustrate the reposition of base pairs that flank the cleavage site upon the binding of different drugs. The etoposide-, *m*-AMSA- and mitoxantone-bound structures are colored in blue, yellow and cyan, respectively. (**B**-**D**) Stereo representation of the DNA cleavage site of the etoposide- (**B**), *m*-AMSA- (**C**) and mitoxantone-bound (**D**) hTop2β^{core} cleavage complexes to show the interaction networks around the −1 nucleotide. For each structure, dashed black lines indicate interactions common to all three structures; dashed red lines indicate interactions unique to the *m*-AMSA- and mitoxantrone-bound structures. Red spheres stand for water molecules. Dashed green lines illustrate the octahedral coordination around the active-site divalent magnesium (green sphere).

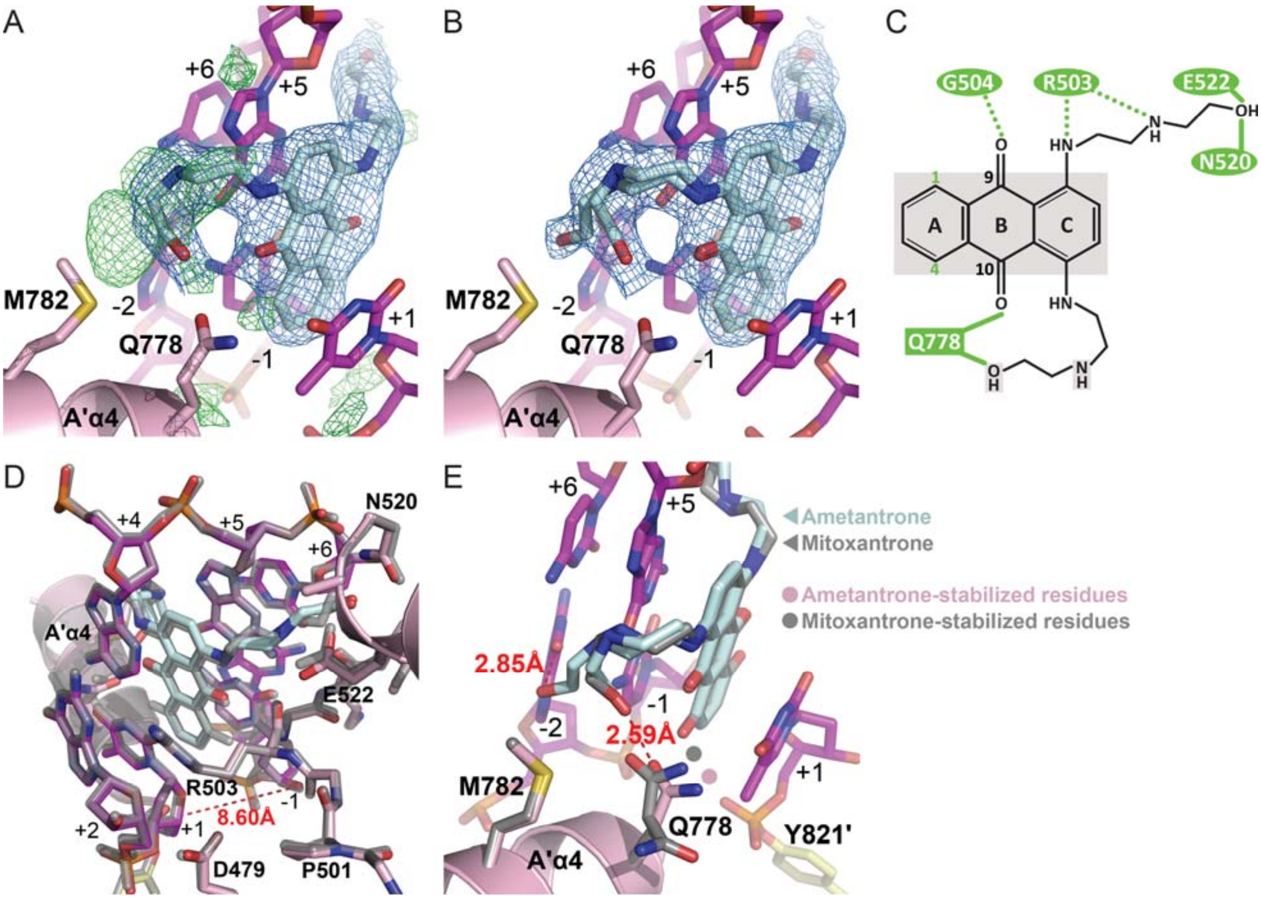

Figure S5. Ametantrone exhibits an extremely similar interaction network but reduced stabilizing force with hTop2β cleavage complex compared to its analog mitoxantrone. (**A**) The 2mF_o-DF_c (blue meshes; contoured at 1.0σ) and unbiased mF_o-DF_c (green meshes; contoured at 3.0σ) difference electron density maps of bound ametantrone after building one drug molecule into the structure at one DNA cleavage site. The strong positive peak in the mF_o-DF_c difference map at the major groove-binding pocket indicates a dual conformation for one alkylamino arm of ametantrone. (**B**) The 2mF_o-DF_c electron density maps (blue meshes; contoured at 1.0σ) of bound ametantrone after building the dual conformation of the drug. (**C**) Chemical structure of ametantrone. Drug-contacting residues are indicated. The interactions mediated by side-chain and main-chain atoms are shown as green solid and dashed lines, respectively. Atoms involved in drug-DNA interactions are shaded in gray. (**D**-**E**) Superposition of the hTop2β^{core}-DNA-mitoxantrone and hTop2β^{core}-DNA-ametantrone ternary complexes demonstrates structural differences between the minor groove- and major groove-binding pockets, respectively. DNA bases of the hTop2β^{core}-DNA-ametantrone ternary complexes are shown as purple sticks, and the protein is shown using a

cartoon/stick representation and colored in pink and yellow according to the chain. The entire structure of the hTop2β^{core}-DNA-mitoxantrone complex is shown in gray. Labels belonging to the second monomer are flagged by a prime. The distance between the Y821'-linked scissile phosphate and the 3'-OH of the ametantrone-bound structure is indicated in panel **D**. Interactions between the dual-terminal hydroxyl groups of the alkylamino arm of ametantrone and the protein are indicated in panel **E**.

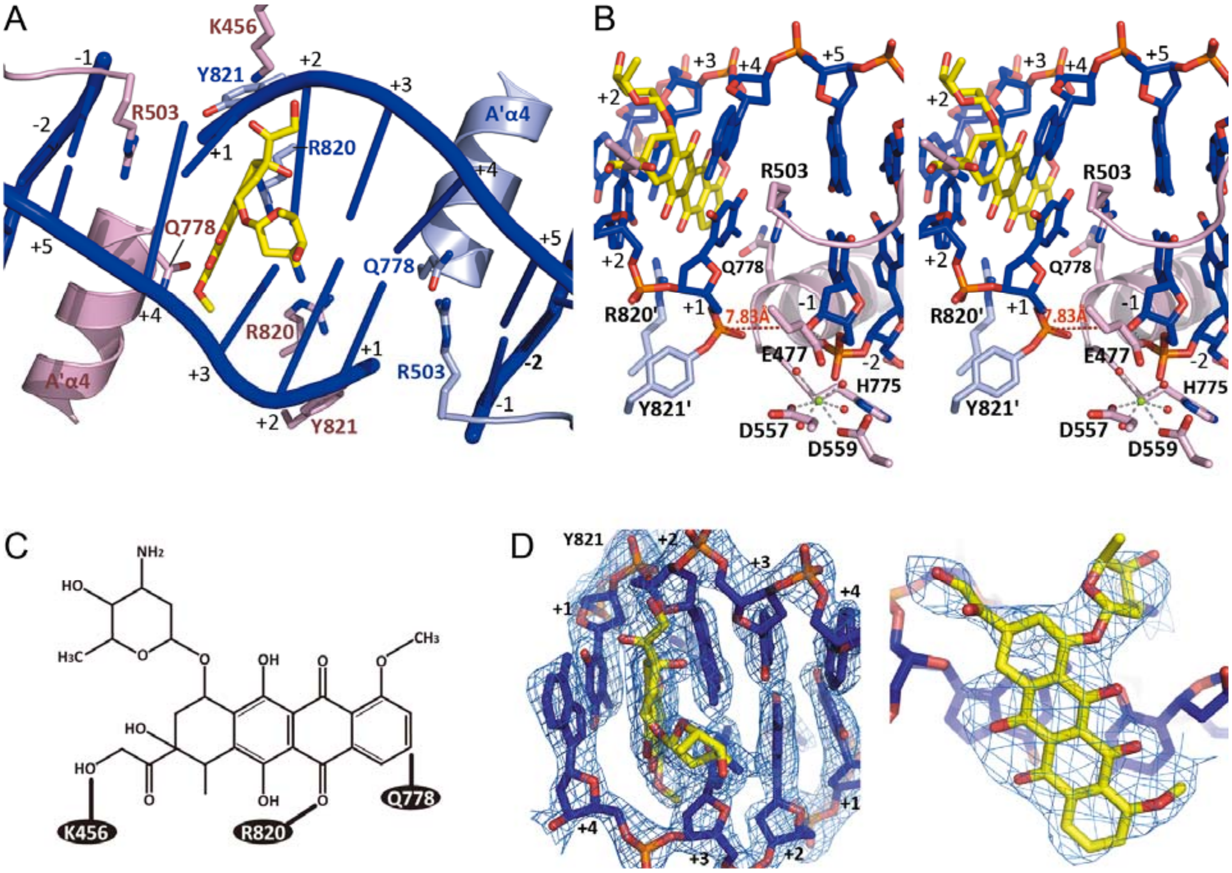

Figure S6. Structural details of the doxorubicin-bound hTop2β^{core} cleavage complex. (**A**-**B**) Cartoon/stick (left) and close-up stereo (right) representations show the targeting sites of doxorubicin. DNA is shown in blue and the two hTop2β protomers are colored differently. Mg^{2+} and water molecules are shown as green and red spheres, respectively. The distances between the Y821-linked scissile phosphate and the 3'-OH are indicated. (**C**), Chemical structures of doxorubicin. Drug-interacting residues are indicated. The interactions mediated by side chain and main chain atoms are shown as solid and dashed lines, respectively. (**D**) The final 2mF_o-DF_c maps (left panel; contoured at 1.2σ) show the electron densities around the binding sites of doxorubicin. The unbiased mF_o-DF_c electron density maps (contoured at 2.8σ) for the bound anticancer drugs are shown in the right panel.

SUPPLEMENTARY TABLE

**Structure**	hTop2β^{core}-DNA-doxorubicin ternary complex
**Space group**	$P2_1$
**Unit cell dimensions**	
a, b, c (Å)	80.3, 176.6, 94.0
β (degrees)	111.5
**Data collection**	
Wavelength (Å)	0.97622
Resolution (last shell)[a] (Å)	30.0-2.68 (2.73-2.68)
Observed reflections	529470
Unique reflections	68785
Completeness (last shell)[a] (%)	100.0 (100.0)
Multiplicity	7.7
Mean 〈$I/\sigma I$〉 (last shell)[a]	12.4 (4.9)
$R_{sym}{}^{b}$ (last shell)[a] (%)	0.09 (0.47)
**Refinement**	
Resolution range (Å)	28.64-2.68
No. of reflection in working set (test set)	67109 (3383)
$R_{\mathrm{crys}}{}^{c}$ (%)	0.16
$R_{\mathrm{free}}{}^{c}$ (%)	0.21
**r.m.s. deviation from ideal**	
Bond lengths (Å)	0.007
Bond angles (degrees)	0.118
**Ramachandran analysis**[d]	
Outliers (%)	0.2
Favored (%)	96.0

Table S1. Crystallographic analysis of hTop2β^{core}-DNA-doxorubicin ternary complex.

[a]Statistics for data from the resolution shell of 2.73-2.68 Å.

[b]$R_{\mathrm{sym}} = (\Sigma|Ihkl - \langle I \rangle)/(\Sigma Ihkl)$, where the average intensity〈I〉is taken overall symmetry equivalent measurements, and *Ihkl* is the measured intensity for any given reflection.

[c]$R_{\mathrm{cryst}} = (\Sigma||F_o| - k|F_c||)/(\Sigma|F_o|)$. $R_{\mathrm{free}} = R_{\mathrm{cryst}}$ for a randomly selected subset (5%) of the data that were not used for minimization of the crystallographic residual.

[d]Categories were defined by PHENIX (5). All non-glycine residues are included for this anlaysis. Although D1101 of both chains in the structure fell in the disallowed region of the Ramachandran plot, these residues fit very well to the corresponding electron density.

SUPPLEMENTARY REFERENCES

1. Wu, C.C., Li, T.K., Farh, L., Lin, L.Y., Lin, T.S., Yu, Y.J., Yen, T.J., Chiang, C.W. and Chan, N.L. (2011) Structural basis of type II topoisomerase inhibition by the anticancer drug etoposide. *Science*, **333**, 459-462.
2. Minotti, G., Menna, P., Salvatorelli, E., Cairo, G. and Gianni, L. (2004) Anthracyclines: molecular advances and pharmacologic developments in antitumor activity and cardiotoxicity. *Pharmacol Rev*, **56**, 185-229.
3. Howerton, S.B., Nagpal, A. and Williams, L.D. (2003) Surprising roles of electrostatic interactions in DNA-ligand complexes. *Biopolymers*, **69**, 87-99.
4. Capranico, G., Kohn, K.W. and Pommier, Y. (1990) Local sequence requirements for DNA cleavage by mammalian topoisomerase II in the presence of doxorubicin. *Nucleic Acids Res*, **18**, 6611-6619.
5. Adams, P.D., Afonine, P.V., Bunkoczi, G., Chen, V.B., Davis, I.W., Echols, N., Headd, J.J., Hung, L.W., Kapral, G.J., Grosse-Kunstleve, R.W. *et al.* (2010) PHENIX: a comprehensive Python-based system for macromolecular structure solution. *Acta Crystallogr D Biol Crystallogr*, **66**, 213-221.
